# Haematogenous cytokeratin 20 mRNA as a predictive marker for recurrence in oral cancer patients

**DOI:** 10.1038/sj.bjc.6690377

**Published:** 1999-05-01

**Authors:** H Kawamata, D Uchida, K Nakashiro, S Hino, F Omotehara, H Yoshida, M Sato

**Affiliations:** Second Department of Oral and Maxillofacial Surgery, Tokushima University School of Dentistry, Tokushima 770, Japan

**Keywords:** oral cancer, CK20, recurrence, metastasis

## Abstract

We examined the expression of cytokeratin 20 (CK20) mRNA in the peripheral blood of oral squamous cell carcinoma (SCC) patients by reverse transcriptase polymerase chain reaction (RT-PCR). Eleven out of 12 oral SCC patients showed positive RT-PCR results. However, there is no clear relationship between the haematogenous CK20 mRNA and the metastasis. After initial treatment, all of the tumour-free survivors tested showed negative RT-PCR results. CK20 mRNA in peripheral blood can be used as a marker for tumour recurrence but not for metastasis in oral SCC patients. © 1999 Cancer Research Campaign


British Journal of Cancer (1999) **80**(3/4), 448–452
© 1999 Cancer Research Campaign
Article no. bjoc.1998.0377

Short communication

### Haematogenous cytokeratin 20 mRNA as a predictive marker for recurrence in oral cancer patients

**H Kawamata, D Uchida, K Nakashiro, S Hino, F Omotehara, H Yoshida and M Sato**

Second Department of Oral and Maxillofacial Surgery, Tokushima University School of Dentistry, Tokushima 770, Japan

**Summary** We examined the expression of cytokeratin 20 (CK20) mRNA in the peripheral blood of oral squamous cell carcinoma (SCC) patients by reverse transcriptase polymerase chain reaction (RT-PCR). Eleven out of 12 oral SCC patients showed positive RT-PCR results. However, there is no clear relationship between the haematogenous CK20 mRNA and the metastasis. After initial treatment, all of the tumour-free survivors tested showed negative RT-PCR results. CK20 mRNA in peripheral blood can be used as a marker for tumour recurrence but not for metastasis in oral SCC patients.

**Keywords:** oral cancer; CK20; recurrence; metastasis

Oral squamous cell carcinoma (SCC) are characterized by a high degree of local invasiveness and a high rate of metastasis to cervical lymph nodes, but a low rate of metastases to distant organs. To successfully establish a metastasis, tumour cells must leave the primary site, invade the local host tissue, enter the circulation (intravasation), arrest at the distant vascular bed, extravasate and proliferate at the distant organ site (Fidler, 1990; Liotta and Stetler-Stevenson, 1991; Aznavoorian et al, 1993). Therefore, if we can evaluate the capability of the tumour cells for intravasation, extravasation and survival in the circulation, we might predict the metastatic potential of the tumour cells in oral SCC patients. In the present study, we examined the expression of cytokeratin 20 (CK20) mRNA in the peripheral blood by reverse transcriptase polymerase chain reaction (RT-PCR) to see the accomplishment of the intravasation of cancer cells and the survival of cancer cells in the circulation.

### PATIENTS, MATERIALS AND METHODS

### Patients

We studied 12 patients. Site of origin, TNM classification at first visit, histopathological diagnosis, lymph node metastasis, and survival periods of the patients are shown in Table 1. Fourteen blood samples from healthy volunteers and three patients with inflammatory lesions of the oral mucosa were subjected to the study.

### Cells and cell culture

BHY, HN, HNt (Kawamata et al, 1997) and IH (Harada et al, unpublished data) cells are all derived from human oral SCC tumours from the patients. TYS is a human oral adenosquamous carcinoma cell line established in our laboratory (Yanagawa et al, 1986). Gingival epithelial cells (GE) and gingival fibroblasts (GF) are cultured from a normal human gingival tissue. All of the cells were maintained in Dulbecco's modified Eagle medium (DMEM) (Gibco BRL, Gaithersburg, MD, USA) supplemented with 10% fetal calf serum (FCS) (Gibco), 100 μg $ml^{-1}$ streptomycin, and 100 U $ml^{-1}$ penicillin (Gibco) in a humidified atmosphere of 95% air and 5% carbon dioxide at 37°C.

### Blood samples

Venous blood (5 ml) from patients and healthy volunteers was collected by puncturing the skin with a Jelco i.v. catheter (Johnson & Johnson Medical, Tokyo), which consisted of an inner metal needle and an outer elastic catheter. To avoid the contamination of the blood with skin fragments, the first 1 ml of blood samples drawn from the inner needle were discarded with the needle. Subsequently, blood samples (5 ml) drawn from the outer catheter were collected in the tubes containing 5 mg EDTA. Twenty millilitres of a haemolytic buffer (10 mM potassium bicarbonate, 155 mM ammonium chloride, 0.1 mM EDTA) was added to the blood samples. Thirty minutes after incubation on ice, the tubes were centrifuged at 3000 ***g*** for 10 min at room temperature. The haemolytic reaction was repeated three times. The pellets were washed with 100 mM phosphate-buffered saline (pH 7.4) twice, and were subjected to the RNA extraction procedure. RNA was extracted by NP-40 hypotonic method (Kawamata et al, 1997) or by Isogen RNA extracting mixture (Nippon Gene, Toyama, Japan). When intact 28S and 18S ribosomal RNAs in the extracted total RNA could be visualized on formaldehyde denaturing agarose gel, 5 μg of total RNAs from the samples were subjected to RT-PCR.

### Reverse transcriptase polymerase chain reaction

Five micrograms cytoplasmic or total RNA was reverse transcribed by Molony Murine Leukemia Virus reverse transcriptase (Gibco) at 42°C for 60 min using random primer (5 μM; Gibco) in 20 μl of the reaction mixture. Subsequently, 1 μl of the product was subjected to the PCR amplification. PCR was performed as

*Received 27 November 1997*
*Revised 15 June 1998*
*Accepted 29 July 1998*

*Correspondence to:* H Kawamata

**Table 1** Site of origin and clinical details of oral SCC patients

| Patients | Site of origin | TNM | pTNM | Primary or recurrent | Histopathology of original tumour | Clinical details | |
|---|---|---|---|---|---|---|---|
| | | | | | | Metastasis[a] (lymph node) | Survival |
| P1 | Tongue | T4N0M0 | pT4N0MX | Recurrent | Invasive SCC | No | Dead (11m[b]) |
| P2 | Mandibular gingiva | T3N1M0 | pT3NXMX | Primary | Invasive SCC | ND | Alive (1 y 2 m) |
| P3 | Tongue | T1N0M0 | pT1N2MX | Primary | Invasive SCC | Yes | Alive (1 y 7 m) |
| P4 | Floor of mouth | T2N0M0 | pT2N1MX | Primary | Basaloid SCC | Yes | Alive (1 y 2 m) |
| P5 | Mandibular gingiva | T2N0M0 | pT2N1MX | Primary | Invasive SCC | Yes | Alive (1 y 4 m) |
| P6 | Mandibular gingiva | T2N0M0 | pT2N0MX | Recurrent | Early invasive SCC | No | Dead (1 y 8 m) |
| P7 | Maxillary gingiva | T2N0M0 | pT2N0MX | Primary | Invasive SCC | No | Alive (10 m) |
| P8 | Tongue | T3N0M0 | pT2N0MX | Primary | Early invasive SCC | No | Alive (9 m) |
| P9 | Tongue | T1N0M0 | pT2NXMX | Primary | Early invasive SCC | ND | Alive (7 m) |
| P10 | Maxillary gingiva | T3N1M0 | pT3NXMX | Primary | Invasive SCC | NI | Alive (7 m) |
| P11 | Mandibular gingiva | T2N2aM0 | pT2N0MX | Recurrent | Invasive SCC | No | Alive (2 y 11 m) |
| P12 | Maxillary gingiva | T2N0M0 | pT2N1MX | Recurrent | Invasive SCC | Yes | Dead (2 y 5 m) |
| P13 | Soft palate | T3N2M0 | pT3N1MX | Primary | Invasive SCC | Yes | Alive (1 y 8 m) |
| P14 | Tongue | T2N1M0 | pT2N0MX | Primary | Invasive SCC | No | Alive (2 y 5 m) |
| P15 | Mandibular gingiva | T3N1M0 | pT3NXMX | Primary | Invasive SCC | NI | NI |
| P16 | Buccal mucosa | T2N0M0 | pT2NXMX | Primary | Invasive SCC | ND | Alive (1 y 7 m) |

[a]Yes, lymph node metastasis was confirmed by histopathology; No, lymph node metastasis was not detected in the operation materials by histopathology; ND, metastasis was not detectable clinically in the observed periods; NI, not informative. [b]Observed periods from the first visit (y, years; m, months).

follows: the final concentration of dNTPs and primers in the reaction mixture was 200 μM and 1 μM respectively. *Taq* DNA polymerase (Takara, Otsu, Japan) was added to the mixture at a final concentration of 0.05 U $\mu l^{-1}$ and the reaction was carried out in Takara Thermal Cycler MP (Takara). The PCR condition was as follows: for glyceraldehyde phosphate dehydrogenase (GAPDH) amplification, 94°C for 3 min and then 94°C for 1 min, 55°C for 1.5 min, 72°C for 2.5 min for 20 cycles and an extension of 72°C for 4 min; for CK20 amplification from cultured cells or oral SCC tissues, 94°C for 3 min and then 94°C for 1 min, 60°C for 1.5 min, 72°C for 2.5 min for 40 cycles and an extension of 72°C for 4 min. For CK20 amplification from blood samples, two-round PCR was used. The conditions were as follows: 94°C for 3 min and then 94°C for 1 min, 65°C for 1.5 min, 72°C for 2.5 min for 30 cycles and an extension of 72°C for 4 min, and then 1 μl aliquots of first-round PCR products were subjected to the second amplification using the same primers and the same conditions. The primers used were: CK20-UP, 5′-CAGACACACGGTGAACTA-3′ (Burchill et al, 1995*a*), for CK20-DN, 5′-GATCAGCTTCCACTGTTAGAC-3′ (Buchill et al, 1995*a*) for CK20 amplification; GAPDH-UP, 5′-GAAATCCCATCACCATCTTCCAGG-3′, and GAPDH-DN, 5′-CATGTGGGCCATGAGGTCCACCAC-3′ for GAPDH amplification.

### Cell spiking

HNt cells (10–1000) were added to 5 ml of whole blood samples from a healthy male volunteer, total RNA was extracted from the cell-spiked blood samples, and RT-PCR for CK20 mRNA was performed.

### Southern blot analysis

RT-PCR products were separated on 1% agarose gels and Southern blot analysis was carried out by the standard method. The probe used was a digoxigenin-labelled oligonucleotide probe CK20-PR (5′-ATACTTCAGTCTGAAGTCCTCAGCAGCCAG-3′), the sequence of which lay between a pair of amplifying primers CK20-UP and CK20-DN.

### Ethical standards

This study has been approved by the ethical committee of Tokushima University School of Dentistry. The patients gave their informed consent before their inclusion in this study.

### RESULTS

### Site of origin and clinical details of oral SCC patients

The clinicopathological details of the patients are shown in Table 1. Twelve blood samples from P1 to P12, and seven fresh cancer samples from P4, P6, P12 and P13–P16 were available.

### RT-PCR detection of CK20 mRNA in human oral SCC cells and human oral SCC tissues

RT-PCR for CK20 mRNA generated a 370-bp band on agarose gel, but some non-specific extra bands appeared on TYS and HNt cells (Figure 1). Southern blot analysis revealed that only a 370-bp band was hybridized with the CK20 specific probe (data not shown). CK20 mRNA was expressed in all of the oral SCC cells

© Cancer Research Campaign 1999 

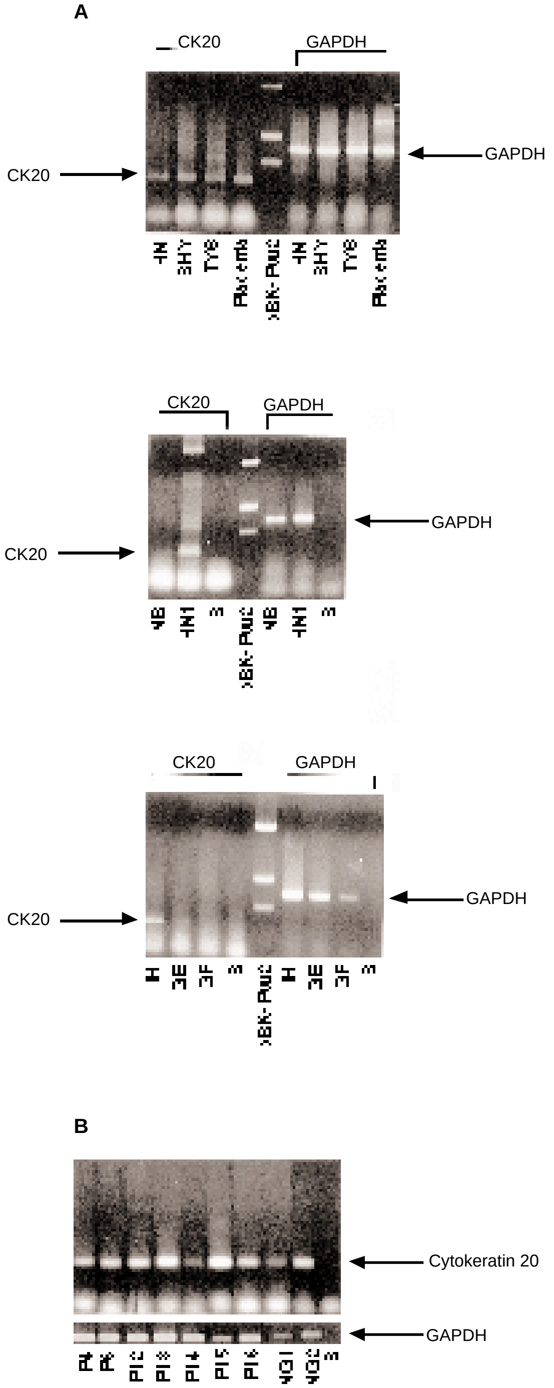

**Figure 1** Amplification of CK20 mRNA from several human oral SCC cell lines (**A**) and human oral SCC tissues (**B**) by RT-PCR. Five micrograms cytoplasmic RNA was reverse-transcribed by M-MLV, and 1 µl of product in a 20-µl reaction was subjected to the PCR amplification. PCR products were separated on 1.5% agarose gels, and stained with ethidium bromide. HN, BHY, HNt, IH: human oral SCC cell lines; TYS: human adenosquamous cell line; placenta: normal placental tissue; NB: normal blood sample; GE: gingival epithelial cells; GF: gingival fibroblasts; B: blank (no RNA); P4, P6, P12, P13–P16: oral cancer tissues; NG1 and NG2: normal gingiva; pBK-Pvu2: molecular weight marker; Pvu 2-digested pBK-CMV plasmid (2878, 1026, 608 bp)

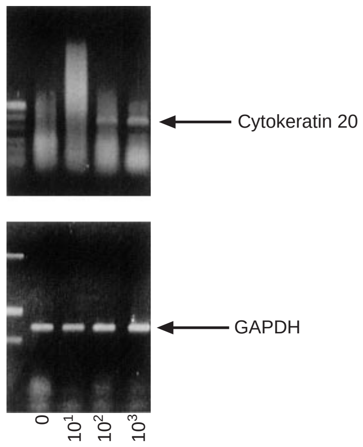

**Figure 2** Amplification of CK20 mRNA from blood samples spiked with HNt cells by RT-PCR. HNt cells (10–1000) were added to 5 ml of whole blood samples from a healthy male volunteer, total RNA was extracted from the cell-spiked blood samples, and 5 µg of total RNA was subjected to RT-PCR. $10^1$, $10^2$, $10^3$: HNt cell number which was added to the blood samples. Molecular weight markers (2878, 1026, 608 bp) were loaded on the first lane

(HN, BHY, HNt, IH), one of the salivary adenosquamous cancer cells (TYS), placental tissue and gingival epithelial cells (GE), but not in peripheral blood cells collected from a healthy man (NB) and gingival fibroblasts (GF) (Figure 1A). Moreover, CK20 mRNA was expressed in all of the oral SCC tissues (Figure 1B). Although CK20 fragments could be amplified by 25–30 cycles with 55°C annealing temperature, some extra bands appeared on the gel. Therefore, we used the condition described in the Materials and methods section (40 cycles with 60°C annealing temperature) to obtain the clear band. GAPDH as an internal control was amplified in all of the samples tested (Figure 1). No amplification was observed in water control samples (B) (Figure 1).

### Control blood analysis and cell spiking

In 17 control blood samples collected from 14 healthy volunteers and three patients with inflammatory lesions of the oral mucosa, CK20 mRNA was not amplified after two rounds of PCR, however GAPDH mRNA was clearly amplified in all of the blood samples after one round of PCR (data not shown). To test the sensitivity of the technique to detect circulating cancer cells in peripheral blood, a cell spiking experiment was performed. We could detect down to $1 \times 10^2$ HNt cells diluted in 5 ml of whole human blood collected from a healthy man (Figure 2). The 370-bp band on agarose gel electrophoresis was confirmed as the CK20 fragment by Southern blot analysis (data not shown). Southern blot analysis did not increase the sensitivity of the assay, but increased the specificity (data not shown). In this PCR reaction, we also used high annealing temperature (65°C) and high cycle numbers (30 plus 30 cycles) to clearly amplify the specific band and to diminish the extra bands. Nested-PCR may be more suitable to amplify such a small amount of mRNA specifically, however we obtained the specific band only in the cell spiking samples but not in the control samples by this method and the band was confirmed as the CK20 fragment by Southern blot analysis. We,

 © Cancer Research Campaign 1999

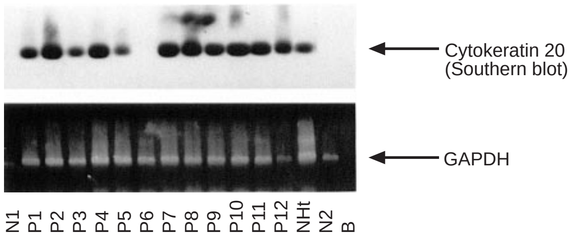

**Figure 3** Amplification of CK20 mRNA from blood samples collected from oral SCC patients by RT-PCR. Total RNA was extracted from 5 ml blood samples from oral SCC patients, and 5 µg of total RNA was subjected to RT-PCR. PCR products were separated on 1.5% agarose gels, and stained with ethidium bromide for GAPDH, or were transferred onto nylon filters and hybridized with digoxigenin-labelled oligonucleotide probe for CK20. P1–P12: oral SCC patients; N1 and N2 healthy volunteers; HNt: oral SCC cells; B: blank (no RNA)

therefore, believed that our technique used in this experiment detected the CK20 mRNA as specifically as nested-PCR did.

### Detection of CK20 mRNA in the blood samples collected from oral SCC patients

Contrary to our expectation, we detected CK20 mRNA in 11 out of 12 oral SCC patients, including eight primary cancer patients and three recurrent patients in their peripheral blood samples (Figure 3). However, there is no clear relationship between the haematogenous CK20 mRNA and cancer metastasis of oral SCC patients. Only one recurrent patient (P6) who had multiple but early invasive SCC on the mandibular gingiva showed negative results for CK20 mRNA amplification in her peripheral blood sample (Figure 3). GAPDH was clearly amplified in all of the samples tested, and no amplification was observed in water control samples (Figure 3). We examined tumour-free survivors (P2, P4, P5, P7, P8 and P9) for CK20 mRNA again in their peripheral blood, 3–16 months after initial treatment (radiation, chemotherapy and operation). All of the patients tested showed negative RT-PCR results for CK20 mRNA (data not shown). We repeated all of the experiments at least three times, and obtained the same results.

### DISCUSSION

Most of the oral SCC cells showed muscular invasion, bone invasion and vascular invasion (lymphatic channels and microvessels) at very early stage. We sometimes observed intravascular tumour embolus on the biopsy materials obtained from oral SCC patients. These observations suggest that oral SCC cells may easily intravasate into the circulation. In fact, we have detected CK20 mRNA in peripheral blood samples collected from 11 out of 12 cases of oral SCC patients as evidence for the existence of cancer cells in the circulation. Several groups have reported use of the RT-PCR method to detect prostatic cancer cells (Fadlon et al, 1996; Henke et al, 1997; Melchior et al, 1997), melanoma cell (Foss et al, 1995), and neuroblastoma cells (Burchill et al, 1995*b*) in peripheral blood by amplifying prostatic specific antigen, tyrosinase and tyrosine hydroxylase respectively. Some investigators used the epithelium-specific cytokeratin (CK8, CK19 or CK20) as a marker for detecting epithelial cancer cells which did not have any specific markers (Burchill et al, 1995*a*; Krismann et al, 1995; Soeth et al, 1996). However, Burchill et al (1995*a*) and Krisman et al (1995) reported that CK8 and CK19 were amplified in a high proportion of normal peripheral blood samples, but CK20 was not amplified in any normal blood samples. In contrast, Moll et al (1992) reported that oral mucosa and oral SCC rarely expressed CK20 protein when examined by immunohistochemistry. However, we detected CK20 mRNA in all of the oral SCC tissues as well as oral SCC-derived cell lines tested when examined by RT-PCR and Southern blotting. These discrepancies may be due to the different sensitivity of the method used. Therefore, we decided to use the CK20 mRNA as a marker for oral SCC cells in this experiment. CK20 mRNA was not amplified in the peripheral blood samples collected from healthy volunteers and was detected only in oral SCC patients when we eliminated a contamination of the blood samples with skin fragments as carefully as possible. However, there was no clear relationship between the detection of haematogenous CK20 mRNA and cancer metastasis of the patients. These results suggest that oral SCC cells may easily intravasate and the intravasation of cancer cells was not critical step for metastasis formation of oral SCC, but extravasation or ectopic growth potential of oral SCC cells may be more important for their metastasis formation. We examined CK20 mRNA in oral SCC patients 3–16 months after initial treatment, all of the tumour-free survivors tested showed negative RT-PCR results for CK20 mRNA. Thus, CK20 mRNA in the peripheral blood from oral SCC patients cannot be a marker for metastatic potentials of the cancer cells, but it can be used as a marker for the evidence of tumour recurrence or the existence of the metastatic tumours after initial treatment.

Although the number of samples examined in this experiment is limited at this time, we detected circulating cancer cells in most of the oral cancer-burdened patients, but in none of the tumour-free survivors after treatment. These results suggest that haematogenous cytokeratin 20 mRNA can be a marker for tumour recurrence in oral squamous cell carcinoma patients.

### ACKNOWLEDGEMENTS

This study was supported in part by a Grant-in-aid from the Ministry of Education, Science and Culture of Japan.

### REFERENCES

Aznavoorian S, Murphy AN, Stetler-Stevenson WG and Liotta LA (1993) Molecular aspects of tumor cell invasion and metastasis. *Cancer* **71**: 1368–1383

Burchill SA, Bradbury MF, Pittman K, Southgate J, Smith B and Selby P (1995*a*) Detection of epithelial cancer cells in peripheral blood by reverse transcriptase-polymerase chain reaction. *Br J Cancer* **71**: 278–281

Burchill SA, Bradbury FM, Selby P and Lewis IJ (1995*b*) Early clinical evaluation of neuroblastoma cell detection by reverse transcriptase-polymerase chain reaction (RT-PCR) for tyrosine hydroxylase mRNA. *Eur J Cancer* **31A**: 553–556

Fadlon EJ, Rees RC, Mcintyre C, Sharrard RM, Lawry J and Hamdy FC (1996) Detection of circulating prostate-specific antigen-positive cells with prostate cancer by flow cytometry and reverse transcription polymerase chain reaction. *Br J Cancer* **74**: 400–405

Fidler IJ (1990) Critical factors in the biology of human cancer metastasis; twenty-eighth GHA Clowes Memorial Award lecture. *Cancer Res* **50**: 6130–6138

Foss AJE, Guille MJ, Occleston NL, Hykin PG, Hungerford JL and Lightman S (1995) The detection of melanoma cells in peripheral blood by reverse transcription-polymerase chain reaction. *Br J Cancer* **72**: 155–159

Henke W, Jung M, Jung K, Lein M, Schlechte H, Berndt C, Rudolph B, Schnorr D and Loening SA (1997) Increased analytical sensitivity of RT-PCR of PSA

© Cancer Research Campaign 1999 

mRNA decreases diagnostic specificity of detection of prostatic cells in blood. *Int J Cancer* **70**: 52–56

Kawamata H, Nakashiro K, Uchida D, Harada K, Yoshida H and Sato M (1997) Possible contribution of active MMP2 to lymph-node metastasis and secreted cathepsin L to bone invasion of newly established human oral-squamous-cancer cell lines. *Int J Cancer* **70**: 120–127

Krismann M, Tod B, Schröder J, Gareis D, Müller KM, Seeber S and Schütte J (1995) Low specificity of cytokeratin 19 reverse transcriptase-polymerase chain reaction analysis for detection of hematogenous lung cancer dissemination. *J Clin Oncol* **13**: 2769–2775

Liotta LA and Stetler-Stevenson WG (1991) Tumor invasion and metastasis: an imbalance of positive and negative regulation. *Cancer Res (suppl.)* **51**: 5054s–5059s

Melchior SW, Corey E, Ellis WJ, Ross AA, Layton TJ, Oswin MM, Lange PH and Vessella RL (1997) Early tumor cell dissemination in patient with clinically localized carcinoma of prostate. *Clin Cancer Res* **3**: 249–256

Moll R, Lowe A, Laufer J and Franke WW (1992) Cytokeratin 20 in human carcinomas; a new histodiagnostic marker detected by monoclonal antibodies. *Am J Pathol* **140**: 427–447

Soeth E, Roder C, Juhl H, Kruger U, Kremer B and Kalthoff H (1996) The detection of disseminated tumor cells in bone marrow from colorectal-cancer patients by a cytokeratin-20-specific nested reverse-transcriptase-polymerase-chain reaction is related to the stage of disease. *Int J Cancer* **69**: 278–282

Yanagawa T, Hayashi Y, Yoshida H, Yura Y, Nagamine S, Bando T and Sato M (1986) An adenoid squamous carcinoma-forming cell line established from an oral keratinizing squamous cell carcinoma expressing carcinoembryonic antigen. *Am J Pathol* **124**: 496–509

*© Cancer Research Campaign 1999*

